# Prognostic value of metabolic variables of [18F]FDG PET/CT in surgically resected stage I lung adenocarcinoma

**DOI:** 10.1097/MD.0000000000007941

**Published:** 2017-09-01

**Authors:** Xiao-Yi Wang, Yan-Feng Zhao, Ying Liu, Yi-Kun Yang, Ning Wu

**Affiliations:** a Department of Diagnostic Radiology; b PET/CT Center; c Department of Thoracic Surgery, National Cancer Center/Cancer Hospital, Chinese Academy of Medical Sciences and Peking Union Medical College, Beijing, China.

**Keywords:** lung adenocarcinoma, metabolic tumor burden, metabolic variables, PET, prognostic

## Abstract

The objective of this study was to assess the prognostic value of metabolic tumor burden measured by positron emission tomography/computed tomography (PET/CT) in patients with stage I lung adenocarcinoma.

We reviewed 127 consecutive patients with pathologically proven stage I lung adenocarcinoma who underwent pretreatment [18F]FDG PET/CT scans in our hospital from 2005 June to 2012 June. The maximum, mean, and peak standardized uptake value (SUV_max_, SUV_mean_, and SUV_peak_), metabolic tumor volume (MTV), total lesion glycolysis (TLG), and computed tomography volume (CTV) were measured. The Kaplan–Meier and Cox proportional hazards model were used with age, gender, TNM stage, clinical stage, histological grade, nodule type, tumor size, and metabolic parameters to predict progression-free survival (PFS). The cut-off point was determined through receiver-operating characteristic curve.

In univariate analysis, the histological grade, nodule type, diameter (cut-off value of 2.0 cm), CTV (6.56 cm^3^), SUV_max_ (3.25 g/mL), SUV_mean_ (1.58 g/mL), SUV_peak_ (1.84 g/mL), MTV (4.80 cm^3^), and TLG (10.40) were significantly associated with PFS (all *P* value < .05). Patients with poorly differentiated adenocarcinoma, solid nodule type, large size, and high metabolic tumor burden were associated with poor prognosis. In multivariate analysis, only histological grade was independent prognostic factors for progression with a *P* value of .005 (RR, 0.355; 95% CI, 0.173–0.728). Among 5 PET/CT metabolic parameters, only MTV was independent prognostic factors for progression with a *P* value of .031 (RR, 1.118; 95% CI, 1.010–1.237).

Histological grade was an independent predictor for progression in patients with stage I lung adenocarcinoma. Among 5 PET/CT metabolic parameters, only MTV was an independent predictor for progression.

## 1 Introduction

TNM stage has been considered the primary prognostic factor and treatment guided for lung cancer. Since stage I nonsmall cell lung cancer (NSCLC) does not have any lymph nodes or distant metastasis, the individual difference is mainly in T stage, that is, the maximum diameter and the location of tumor (the visceral pleura infiltration). However, as UyBico et al^[1]^ and Adebonojo et al^[2]^ reported, tumor and patient specific factors vary even within the same disease stage, creating a heterogeneous population of patients, each with an individual prognosis that requires consideration of patient- and tumor-specific factors for best estimation. Owing to the high heterogeneity, there is a quite limitation in treatment planning and prognosis predicting only based upon TNM stage and pathological type. It has been reported that the patients with resectable disease and poor prognostic variables might benefit from adjuvant therapy.^[3]^ The latest National Comprehensive Cancer Network guidelines^[4]^ define patients at high risk for poorly differentiated cancer, vascular invasion, visceral pleural invasion, tumor size >4 cm, wedge resection, and incomplete lymph node sampling. These patients may be potential candidates for postoperative adjuvant chemotherapy.

Recently, positron emission tomography/computed tomography (PET/CT) have appeared as significant diagnostic imaging systems in clinical medicine. Accurate recognition of cancers in patients by means of PET/CT scanning with 2-deoxy-2-[18F]fluoro-D-glucose ([18F]FDG) has illustrated a need to determine a mode of therapy to achieve better prognoses. The clinical management of cancer patients has improved dramatically with the introduction of clinical PET.^[5]^ Several researches have already proved that maximum standard uptake value (SUV_max_), metabolic tumor volume (MTV), and total lesion glycolysis (TLG) had prognostic and predictive value in NSCLC patients.^[6,7]^ Beside above clinical information, tumor FDG uptake presurgery is very highly correlated with prognosis and may assist with the postsurgery treatment planning.^[3,8]^

Previous studies focused more on advanced stage lung cancer. Few research results can be found in early stage NSCLC.^[8–10]^ The aim of this work is to investigate the prognostic role of metabolic tumor burden parameters in patients with stage I lung adenocarcinoma who underwent surgically resected.

## 2 Materials and methods

### 2.1 Subjects

This retrospective study was approved by the institutional review board with waiver of informed consent. There were 8644 consecutive primary lung malignant neoplasm patients having surgical operation in our hospital from 2005 June to 2012 June, with 4612 adenocarcinomas, and 851 stage I adenocarcinomas within them. A total of 127 patients with stage I adenocarcinoma, who performed [18F]FDG PET/CT scans before surgical operation, were enrolled in this retrospective study. The electronic medical records of all patients were reviewed, including age, gender, surgical approach, histological grade (poor, moderate, and well), TNM stage, and clinical stage. The distribution and nodule density of the tumor was determined by PET/CT. Nonsolid nodule which means ground-glass opacity on CT scans appears as hazy increased opacity of lung, with preservation of bronchial and vascular margins.^[11]^ Part-solid nodule is defined as mixed nonsolid and solid components. According to the nodule density, the lesions were divided into 3 types: nonsolid, part-solid, and solid. All lesions were analyzed by 3 experienced radiologist with 14, 17, and 30 years working experience, respectively. The radiologists defined the tumor types blindly. The disagreement was decided by discussion.

### 2.2 [18F]FDG PET/CT study

[18F]FDG PET/CT was performed using an integrated PET/CT (Discovery ST, GE Healthcare). All patients in this study were scanned on the same PET/CT machine. Patients’ serum glucose was controlled in normal level (120–200 mg/dL) before undergoing PET/CT examination. Patients received 3.70 to 4.44 MBq/kg of [18F]FDG intravenously, followed by a whole body 3-dimensional PET/CT scan 60 to 70 minutes later. The PET images were obtained with 3 minutes acquisition per bed, with slice thickness 3.27 mm. Scan from skull vertex to upper-thigh resulted in an acquisition time of 15 to 18 minutes. All PET images were reconstructed using an iterative algorithm (ordered-subset expectation maximization, OSEM) with CT-based attenuation correction. Spiral CT was performed with a tube voltage of 120 kV, tube current of 150 mA, 3.75 mm slice thickness, and 3.75 mm interval, at 0.8 second per rotation. Breathing-hold chest CT was performed then, with a tube voltage of 120 kV, tube current of 205 mA, slice thickness of 5 and 1.25 mm, with 5 and 0.8 mm interval, respectively, at 0.8 second per rotation.

### 2.3 Automated PET delineation methods and CT delineation method

There were 2 PET delineation methods used according to different nodule types.

**Figure 1:**
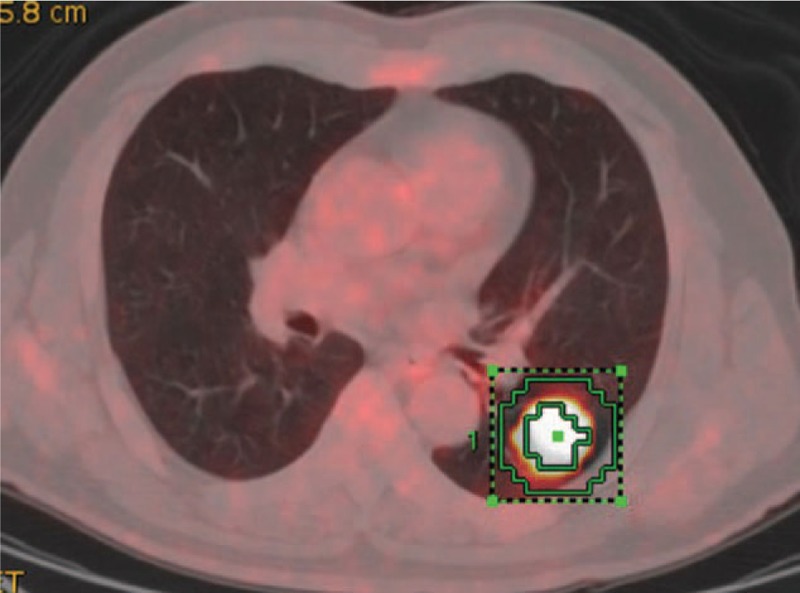
The MTV was segmented using an AT-AIA in PETVCAR. AT-AIA = adaptive iterative algorithm, MTV = metabolic tumor volume, PETVCAR = positron emission tomography volume computerized assisted reporting.

The first method used in solid lesions was adaptive target volume delineation by PET Volume Computerized Assisted Reporting (PETVCAR, GE Healthcare). The PET and CT coregistration was first assessed once the images were loaded into the PETVCAR software. The primary lung cancer PET gray scale and PET/CT fused images were then reviewed in the axial, sagittal, and coronal planes. A boundary box was placed over the image, which was to autocontour and segment the region of interest (ROI), reviewed and adjusted to ensure this 3-dimensional cube contained all the [18F]FDG PET positive area and excluded the negative normal tissue. This process was repeated until each [18F]FDG PET/CT positive region has been selected and optimized. The lesion metabolic volume was automatically segmented using an adaptive iterative algorithm in PETVCAR which separated the target volume from the background tissue by weighting the SUV_max_ and the SUV_mean_ within the target volume with a weighting factor, represented as a Boolean variable^[12]^ (Fig. 1).

**Figure 2:**
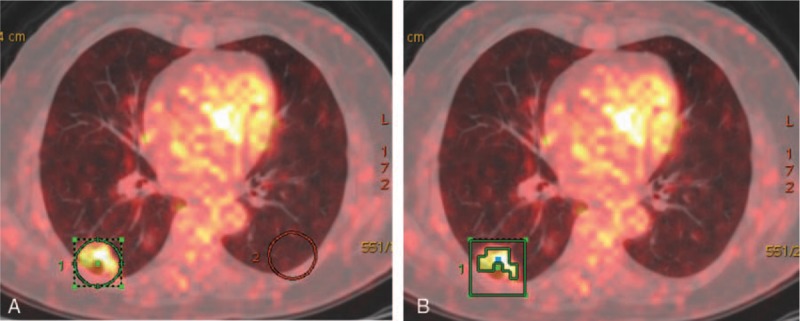
In the method of AT40%, which adapts the threshold value inside the selected VOI relative to mean background (BG) SUV, calculating T value as thresholding, needs the user (A) draw ROI-1 on the slice with SUV_max_ on 2-dimensional images (getting SUV_max_ of lung nodule); copy the ROI-1, select the same location at contralateral lung, then draw the ROI-2 (getting SUV_mean_ of BG). (B) A boundary box of 3-dimensional cube was placed over the image, which was to auto-contour and segment the region of interest using T value as thresholding. AT40% = adaptive thresholding at 40% SUV_max_, BG = background, ROI = region of interest, SUV = standard uptake value, VOI = volume of interest.

The 2nd method used in nonsolid and part-solid lesions was adaptive thresholding at 40% SUV_max_,^[13]^ which adapts the threshold value inside the selected volume of interest relative to mean background (BG) SUV, calculating T value as thresholding: T = 0.4(SUV_max_ − BG) + BG. This delineation method required information on background uptake. The background of lung is heterogeneous; mean background SUV has discrepancy at different region (apex, central, and peripheral region). The user needs to copy the ROI and select the same location at contralateral lung (Fig. 2).

**Figure 3:**
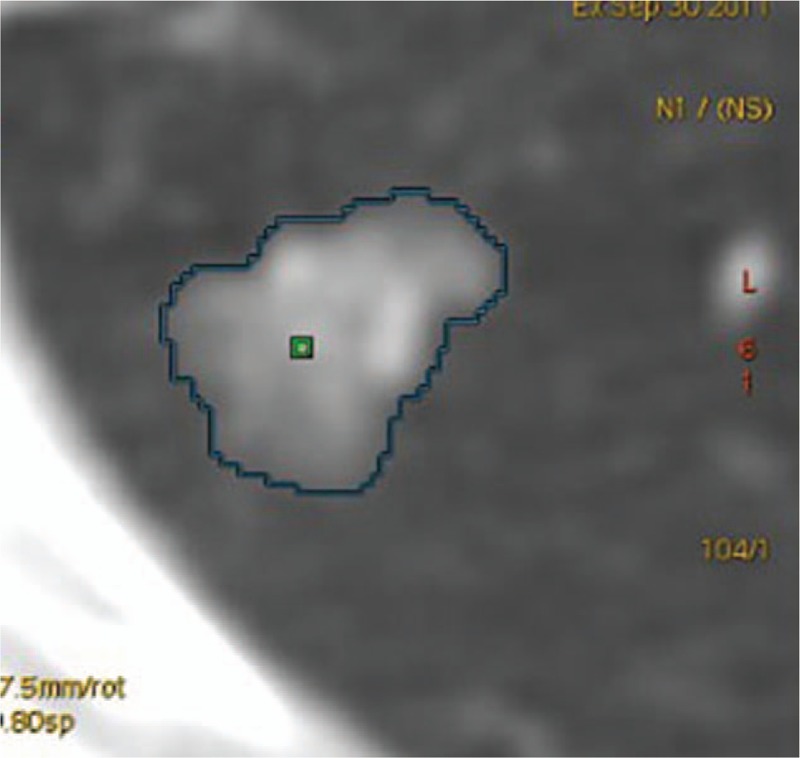
The lesion CTV was measured through Lung VCAR software. CTV = computed tomography volume, Lung VCAR = Lung Volume Computerized Assisted Reporting.

The computed tomography volume (CTV) was measured through Lung Volume Computerized Assisted Reporting software (Lung VCAR software, GE Healthcare) on the 1.25 mm slice thickness images. Lung Volume Computerized Assisted Reporting is an image analysis software package for Advantage Workstation systems that uses GE's Volume Viewer software. The analysis mode was used which offers a combination of 2D reformatted views with correlated volume rendering views, centered on the bookmarked spot. In this mode, the software zooms on the volume of interest, automatically calculates the volume of the suspicious spot, and displays the calculated volume on the views. Also, depending on the protocol chosen, it displays the consistency of the detected nodules. The actual volume measurement is done using an automatic nodule sizing algorithm. By repeated measurements over time, this metric can be used to assess the evolution in volume of a nodule (Fig. 3).

### 2.4 Statistical analysis

Continuous data were expressed as mean ± SD, and categorical data were presented as frequency. Continuous data analysis was performed using the independent *t* test for parametric variables. Multiple groups were compared using ANOVA, which pairwise comparisons using LSD method. Nonparametric variables analysis was performed using the Mann–Whitney test. Categorical data were calculated using Pearson Chi-square test.

Progression-free survival (PFS) was compared by employing the Kaplan–Meier method and Cox proportional-hazard model. The cut-off point of each continuous parameter was determined through receiver-operating characteristic curve and Youden index, using PFS as the classification status. The Kaplan–Meier was used for univariate analysis. The Cox proportional-hazard model was used for multivariate analysis. *P* < .05 was considered statistically significant. The SPSS 13.0 statistics software was used for statistical analysis.

The correlation of each 2 parameters was calculated and compared. The correlation coefficient (*R* value) = 0.21–0.40 for the poor consistency, *R* value = 0.41–0.60 for the general consistency, *R* value = 0.61–0.80 for the good consistency, and *R* value = 0.81–1.00 for the excellent consistency. *P* < .05 were assumed to indicate significant differences.

## 3 Results

A total of 127 patients were evaluated (mean age 60 years; 60 males, 67 females). The diameter of 52 lesions are smaller than 2.0 cm (41%), 50 lesions are 2.0 to 3.0 cm (39%), and 25 lesions are larger than 3.0 cm (20%). The patient data were shown in Table 1. The median follow-up time was 5 years (60 months). At the end of the follow-up period, 96 patients (76%) were alive (mean PFS time of 58.7 ± 23.9 months, median of 60 months) and 31 patients (24%) had recurrence (mean PFS time of 21.6 ± 10.5 months, median of 22 months). Recurrence was reported as follows: 4 cases of local recurrence (bronchial stump), 11 cases of regional recurrence (3 pleural seeding, 2 mediastinal lymph node, and 6 lung metastasis), 12 cases of distant recurrence (3 liver, 5 brain, and 4 bone), and 4 cases with multimetastasis (4 pleural, 3 lymph node, 1 lung, and 1 bone simultaneously).

**Table 1:**
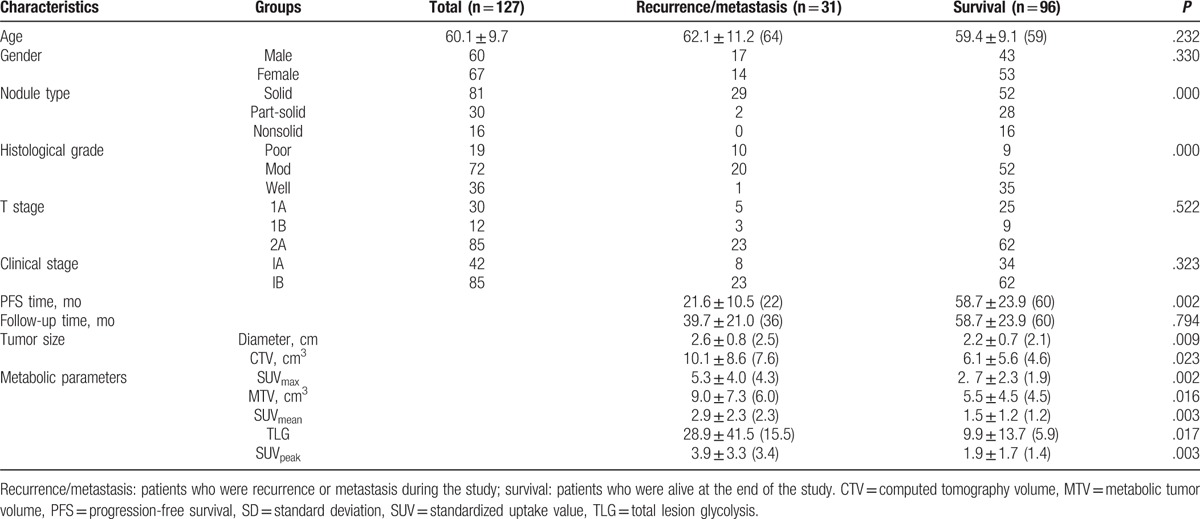
Patient characteristics (mean ± SD, median in the brackets).

**Figure 4:**
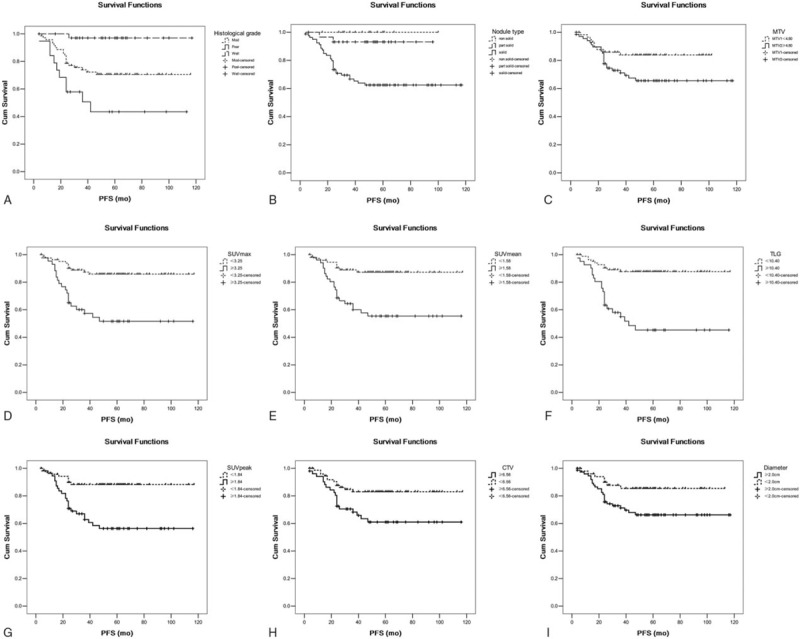
The survival curves of different parameters in Kaplan–Meier univariate analysis. (A) Histological grades (*P* = .000). (B) Nodule types (*P* = .001). (C) MTV with a cut-off value of 4.80 cm^3^ (*P* = .04). (D) SUV_max_ with a cut-off value of 3.25 g/mL (*P* = .000). (E): SUV_mean_ with a cut-off value of 1.58 g/mL (*P* = .000). (F) TLG with a cut-off value of 10.40 (*P* = .000). (G) SUV_peak_ with a cut-off value of 1.84 g/mL (*P* = .000). (H) CTV with a cut-off value of 6.56 cm^3^ (*P* = .009). (I) Diameter with a cut-off value of 2.0 cm (*P* = .022). CTV = computed tomography volume, MTV = metabolic tumor volume, SUV = standard uptake value, TLG = total lesion glycolysis.

The cut-off point of each continuous parameter was determined through receiver-operating characteristic curve and Youden index. In Kaplan–Meier univariate analysis, the histological grade (poor), nodule type (solid), higher MTV (>4.8 cm^3^), SUV_max_ (>3.25 g/mL), SUV_mean_ (>1.58 g/mL), SUV_peak_ (>1.84 g/mL), TLG (>10.40), CTV (>6.56 cm^3^), and diameter (>2.0 cm) were significantly associated with shorter PFS (all *P* < .05). The survival curves of different parameters in Kaplan–Meier univariate analysis were shown in Fig. 4. However, the age, gender, T stage, and clinical stage were not shown to be statistically significant survival prognostic factor in Kaplan–Meier univariate analysis (all *P* > .05).

**Figure 5:**
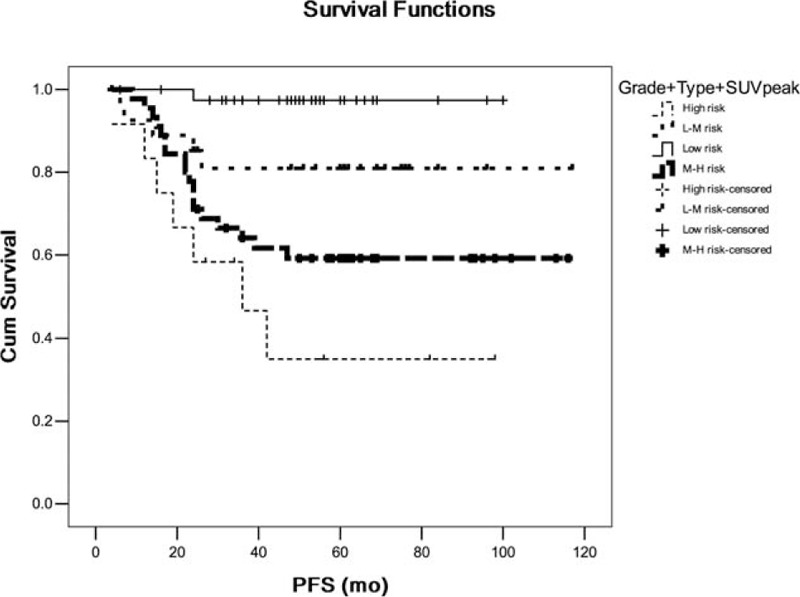
The comparison of patient with poor histological grade as well as solid nodule type and high standard uptake value (SUV)_peak_ versus patient with well grade as well as nonsolid nodule type and low SUV_peak_. Grade: histological grade; L: low risk; M: moderate risk; and SUV_peak_: the peak of standard uptake value. Type: nodule type; low risk: 0 risk factor; low-moderate risk: 1 risk factor; moderate-high risk: 2 risk factors; and high risk: 3 risk factors.

**Figure 6:**
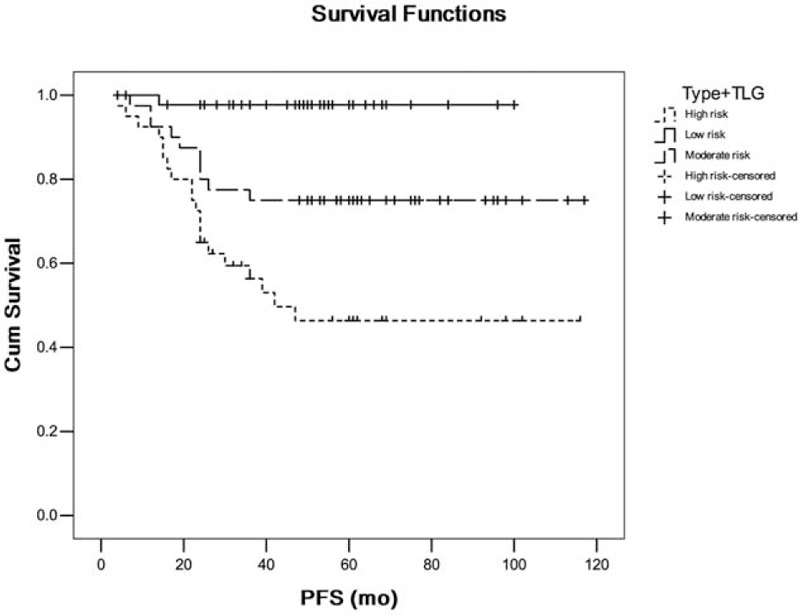
The comparison of patient with solid nodule type as well as high total lesion glycolysis (TLG) versus patient with nonsolid nodule type as well as low TLG. Type: nodule type; low risk: 0 risk factor; moderate risk: 1 risk factor; and high risk: 2 risk factors.

**Figure 7:**
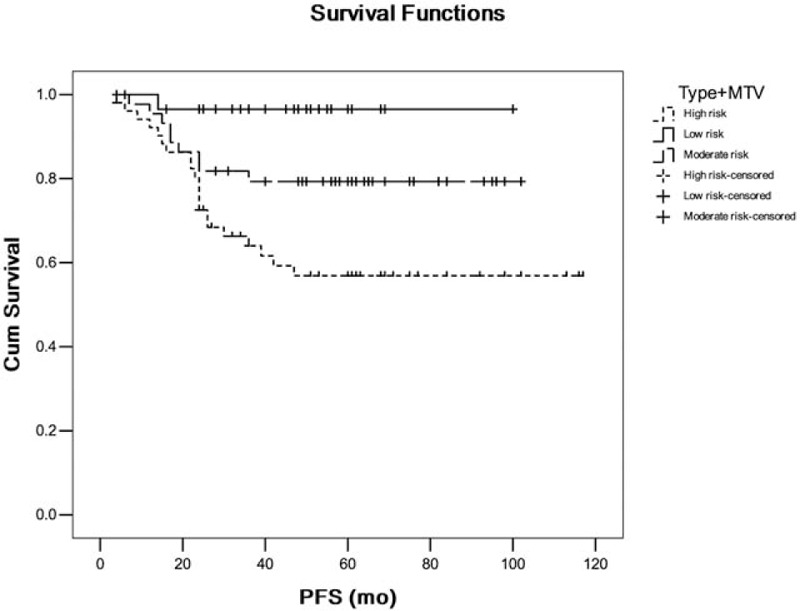
The comparison of patient with solid nodule type as well as high metabolic tumor volume (MTV) versus patient with nonsolid nodule type as well as low MTV. Type: nodule type; low risk: 0 risk factor; moderate risk: 1 risk factor; and high risk: 2 risk factors.

We further identified and analyzed patient groups with overlapping clinical parameter. In Kaplan–Meier survival analysis, patient with poor histological grade as well as solid nodule type and high SUV_peak_ was significantly associated with shorter PFS (*P* < .05) versus patient with well grade as well as nonsolid nodule type and low SUV_peak_ (Fig. 5). Patient with solid nodule type as well as high TLG was significantly associated with shorter PFS (*P* = .000) versus patient with non-solid nodule type as well as low TLG (Fig. 6). Patient with solid nodule type as well as high MTV was significantly associated with shorter PFS (*P* = .001) versus patient with nonsolid nodule type as well as low MTV (Fig. 7).

In Cox proportional-hazard model multivariate analysis, among all different parameters evaluated, only histological grade was shown to be a statistically significant survival prognostic factor with a *P* value of .005 (RR, 0.355; 95% CI, 0.173–0.728). Nodule type showed marginal significance with a *P* value of .053 (RR, 0.227; 95% CI, 0.051–1.021) (Table 2). Among 5 PET/CT metabolic parameters, only MTV was shown to be a statistically significant survival prognostic factor with a *P* value of .031 (RR, 1.118; 95% CI, 1.010–1.237) (Table 3). Among all parameters evaluated excluding histological grade, patient with solid nodule type as well as high TLG was shown to be a statistically significant survival prognostic factor with a *P* value of .019 (RR, 3.416; 95% CI, 1.220–9.565) (Table 4). All other parameters were not independent significant survival prognostic factor (all *P* > .05).

**Table 2:**
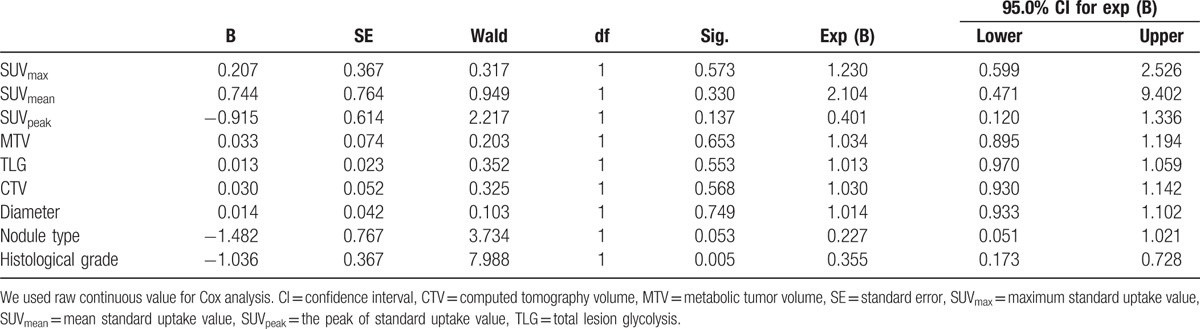
The Cox regression of survival parameters.

**Table 3:**
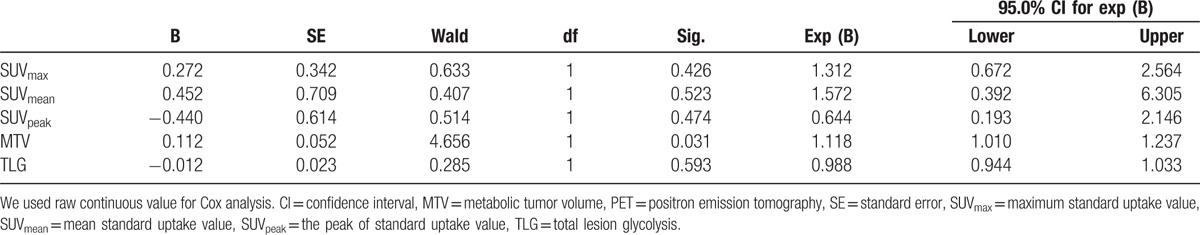
The Cox regression of 5 PET metabolic variables.

**Table 4:**
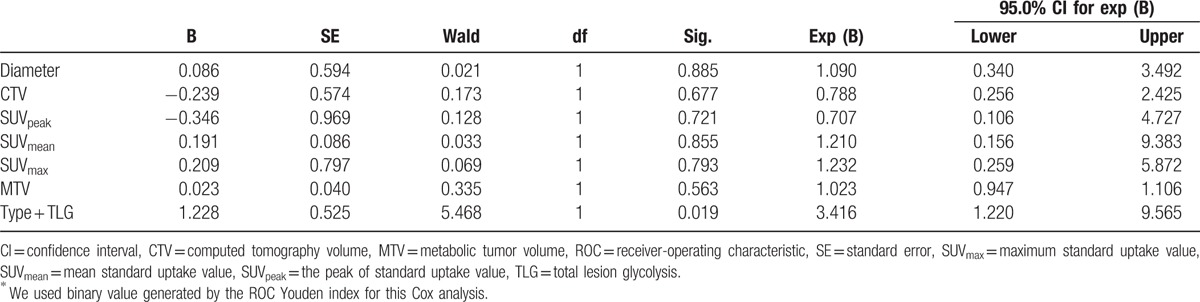
The Cox regression of survival parameters (including overlapping of type + TLG, excluding histological grade)^∗^.

Solid nodule type, poor histological grade, and larger nodule size were associated with higher SUV, MTV, and TLG. Table 5 showed that in the comparison of the parameters among different nodule types, there is statistical significance of SUV_max_, SUV_mean_, SUV_peak_, MTV, and TLG between nonsolid and solid nodules, part-solid and solid nodules (all *P* < .05); but there is no statistical significance between nonsolid and part-solid nodules (all *P* > .05). According to histological grade, there is statistical significance of SUV_max_, SUV_mean_, and SUV_peak_ among poor, mod, and well differentiated nodules (all *P* < .05); but there is no statistical significance of MTV among them (all *P* > .05); TLG had statistical significance between poor and well differentiated nodules, poor and mod differentiated nodules (*P* < .05), but there is no statistical significance between well and mod differentiated nodules (*P* > .05). All of the 5 metabolic parameter had statistical significance between small lesions (<2 cm) and large lesions (≥2 cm) (all *P* = .000).

**Table 5:**
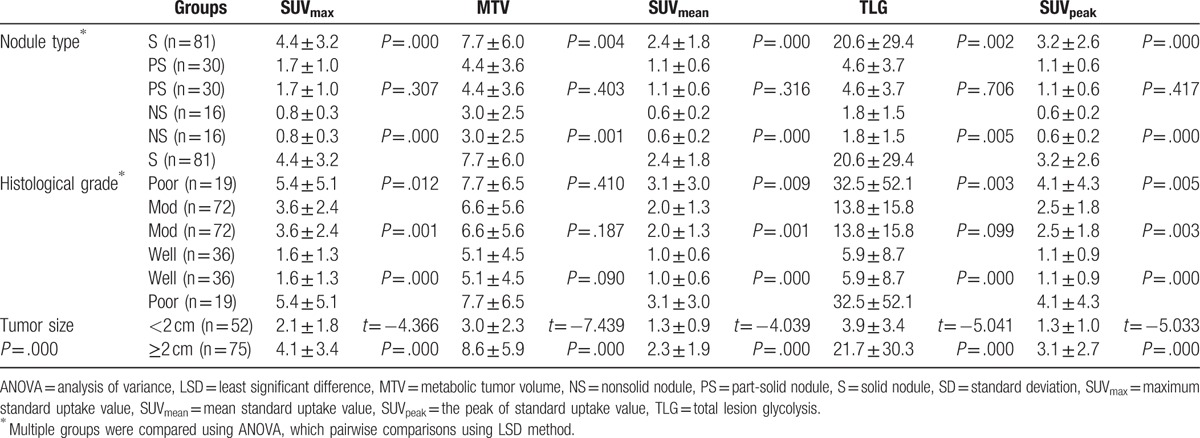
The comparison of the parameters among different patient characteristics groups (mean ± SD, *t*, and *P* value).

Table 6 showed the excellent consistency (*R* value > 0.8) in SUV with MTV and TLG. The nodule types had good consistency with SUV_max_, SUV_peak_, SUV_mean_, and TLG (*R* value > 0.6). The histological grade had general consistency with SUV_max_, SUV_peak_, and SUV_mean_ (*R* value >0.4).

**Table 6:**
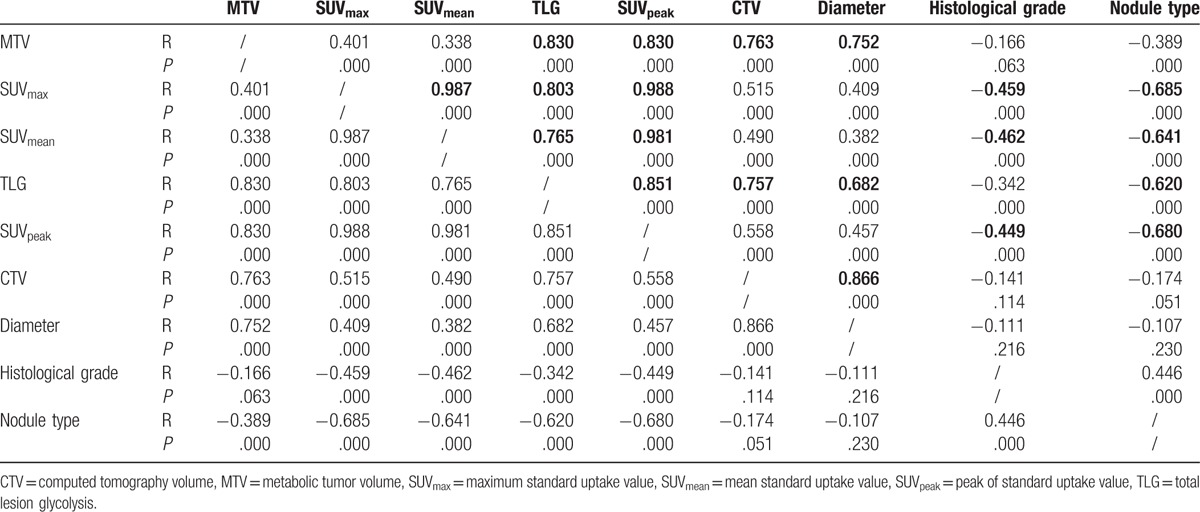
The correlation coefficient of parameter (*R*, *P* value).

## 4 Discussion

The role of PET/CT for NSCLC is not only in diagnosis and differential diagnosis, but also in treatment response evaluation and prognosis prediction. In many common cancers, tumor FDG uptake presurgery is very highly correlated with prognosis and may assist with the postsurgery treatment planning. There were a number of manuscripts on tumor FDG uptake in NSCLC over the recent years demonstrate that FDG uptake level can be used as prognostic indicator. Recent researches showed that SUV is an independent prognostic factor of NSCLC,^[14,15]^ the predictive value of MTV and TLG are controversial.^[6,7,16–20]^

Despite the favorable prognosis of stage IA NSCLC, the disease recurs after complete surgical resection in 20% to 30% of patients.^[3]^ It may due to the high heterogeneity of lung cancer. How to distinguish good prognosis patients from poor prognosis patients is still challenging. It is necessary to predict the prognosis of patients with resectable NSCLC, as patients with poor prognostic variables might benefit from adjuvant therapy.

As reported in the literature, comparing to other histological type of lung cancer, well-differentiated adenocarcinoma was usually with very poor SUV. Those nodules are more difficult in delineating MTV due to the low FDG uptake. The prognostic value of metabolic variables in stage I lung carcinoma is controversial. Yoo et al^[9]^ evaluated the prognostic value of SUV_max_ and MTV in early stage NSCLC patients without lymph node metastasis, considered high SUV_max_ is an independent prognostic factor of shorter DFS. Domachevsky et al^[8]^ evaluated SUV_max_, CT volume, MTV, and TLG as survival prognostic markers in patients with stage I-II NSCLC, considered SUV_max_ is a useful independent prognostic variable. Lin et al^[10]^ aimed to determine the relationship between the preoperative MTV and DFS of patients with stage I NSCLC, considered that SUV_max_ was the only parameter that exhibited an impact on DFS; preoperative MTV parameters have a limited prognostic value for predicting DFS. Park et al^[3]^ aimed to determine the prognostic value of metabolic parameters in surgically resected stage IA NSCLC, considered TLG was a significant prognostic factor for OS, SUV_max_ showed marginal significance.

There have been over the recent years a number of manuscripts on MTV delineation in cancer. Various delineation methods have advantages and limitations. Up to now, it is still challenging to define MTV accurately for small and low uptake lung nodules. In general, well differentiated early stage adenocarcimomas are of lower FDG uptake compared to other histological subtypes of NSCLC. The volumes of well differentiated early stage adenocarcinomas are more difficult segmented due to the very poor SUV_max_ value even in the solid presence. In our study of stage I adenocarcinomas, 80% lung nodules were less than 3 cm and the median SUV_max_ (SUV_mean_) was 4.3(2.3) g/mL in recurrence patients and 1.9 (1.2) g/mL in survival patients. In general, smaller nodules especially are more likely then to have partial voluming effects. Therefore, we used adaptive iterative algorithm by PETVCAR in solid lesions, and adaptive thresholding at 40% SUV_max_ (AT40%) which adapts the threshold value relative to mean background SUV in nonsolid and part-solid lesions. The advantage of the 2 delineation methods was both of them considered the information of background uptake. Adaptive iterative algorithm was usually used on solid nodules larger than 2 cm with high FDG uptake. There is not enough clinical evidence that it is suitable for lesions less than 2 cm with low uptake; it may not be a good method in such a clinical scenario since the gradient of these lesions is usually low. The AT40% method, considering the metabolic contrast between lesion and background uptake and the location of the lesion, may improve the segment accuracy in small and low uptake lesions, although with the manual background ROI procedure, which may introduce more interobserver variability than the other methods. The investigation of the segmentation method on small, low uptake, and heterogeneous lung nodules is still rare. Currently, there is not a universally accepted segmentation method for such a lesion yet.

It is known that a lot of factors have impacts on SUV, such as the body weight, serum glucose level of the patient, and technologic factors (the model of scanning equipment, attenuation correction method and image reconstruction algorithm, radiopharmaceutical activity, injection dose, fasting time, and uptake time). So, the prognostic value of SUV is complicated, although many clinical researches have already shown the correlation of SUV with the patient surviving in NSCLC. SUV_max_ is prone to be affected by the fluctuation caused by the errors in the image. The tumor volume information is ignored when only SUV_max_ is used. According to the research of Melloni et al,^[21]^ this may lead to a significant bias because tumor volume is a prognostic determinant. In addition, SUV measures a single highly metabolic focus that may not reflect the whole tumor metabolic activity.^[16,22]^ So, MTV and TLG have been widely used in the prognostic analysis of NSCLC recently.^[7,23]^

MTV and TLG combined with volume and metabolic information can reflect the characteristics of 2 aspects of the tumor. More precisely, MTV is affected by the size of the tumor and the distribution of the intratumoral SUV. TLG was combined and affected by MTV and SUV_mean_ simultaneously. MTV and TLG are considered to be more reliable indicators of response to tumor burden and aggressiveness than SUV_max_ and tumor size. MTV and TLG can be used to predict the prognosis of various malignancies with heterogeneity. TLG is stated to have a prognostic value in NSCLC. Park et al^[3]^ found that TLG can be used to estimate the resectable region in the operation. Previously, lobectomy and mediastinal lymph node dissection was the standard operational methods. Recently, segmentectomy and wedge resection is more widely used. According to Park et al,^[3]^ stage IA NSCLC patient with high TLG has higher recurrent risk than the patient with low TLG. Therefore, it seems segmentectomy and wedge resection should be avoided on the patient with high TLG. In our study, higher MTV and TLG were significantly associated with shorter PFS (*P* = .04, .000), this result was consistent with Park et al.^[3]^ However, among 5 PET/CT metabolic parameters, only MTV was shown to be a statistically significant survival prognostic factor (RR = 1.118, *P* = .031).

Our study showed that the poor histological grade, solid nodule type, and larger nodule size were associated with higher SUV, MTV, and TLG. The poor histological grade, solid nodule type, higher MTV, SUV_max_, SUV_mean_, SUV_peak_, TLG, larger CTV, and diameter were significantly associated with shorter PFS. The reason should be as follows: first, the FDG uptake will be higher in large lesions because the large lesions contain more active tumor cells than small lesions. Second, malignant tumors will become more aggressive and gain greater malignant potential during growth, that is, evolution, which may lead to a gradual increase in FDG uptake. Kadota et al^[24]^ reported that the SUV_max_ of stage I lung adenocarcinoma was correlated with the histological grading which was developed by IASLC, the American Thoracic Society, and the European Respiratory Society in 2011 year; the poor histological grade was correlated with the high SUV_max_.

Reproducibility evaluation was achieved for the implementation by repeating the delineation procedure several times in each patient. The interobserver variability on the delineation process was less prone to happen because the boundary box was auto-contour and segments the ROI. The user only needs to review and adjust to ensure the 3D-box contained all the FDG positive area and excluded the negative normal tissue. Then the lesion metabolic volume was automatically segmented from the different algorithms. Therefore, we did not do the reproducibility testing to investigate the impact of interobserver variability.

The limitations of this study are as follows. First of all, it is a retrospective study with intrinsic biases. Second, the insufficient numbers of nonsolid and part-solid nodules and the heterogeneous distribution in 3 nodule types maybe cause some biases. Third, we did not investigate the impact caused by the different proportion of solid components within the part-solid nodules.

## 5 Conclusions

In stage I adenocarcinoma the poor histological grade, solid nodule type, higher metabolic parameters, and larger nodule size were significantly associated with shorter PFS. Histological grade was an independent predictor for progression in patients with stage I lung adenocarcinoma. Among 5 PET/CT metabolic parameters, only MTV was an independent predictor for progression. Among all parameters excluding histological grade, patient with solid nodule type as well as high TLG was an independent predictor for progression.
